# Effects of Sow–Piglet Co-Feeding on Post-Weaning Welfare and Jejunal Morphological Development in Suckling Piglets

**DOI:** 10.3390/ani16111690

**Published:** 2026-05-31

**Authors:** Xuanning Liu, Zhihao Zhang, Ying Qian, Yufu Shu, Yameng Li, Zhiyang Zhang, Zhonghui Wang, Sitong Zhou, Honggui Liu, Houjuan Xing

**Affiliations:** 1 College of Animal Science and Technology, Northeast Agricultural University, No. 600 Changjiang Road, Harbin 150030, China; 2 Key Laboratory of Swine Facilities, Ministry of Agriculture and Rural Affairs, Northeast Agricultural University, No. 600 Changjiang Road, Harbin 150030, China

**Keywords:** feeding method, piglets, growth performance, behavior, intestine, diarrhea

## Abstract

Under intensive farming conditions, a decline in feed intake after weaning in suckling piglets often results in reduced body weight or diarrhea. We hypothesized that a sow–piglet co-feeding strategy during the suckling period—in which piglets participate in the sow’s feeding process and consume both lactating sow feed and creep feed—could alleviate certain aspects of weaning stress. To test this hypothesis, 102 newborn piglets (Large White × Duroc × Min Pig) were selected and divided into a co-feeding group (CF) and a non-co-feeding group (NCF), based on whether they had access to the sow’s feed during lactation. The study investigated the effects of the two feeding strategies on piglet growth performance, diarrhea incidence, behavior, and post-weaning immune status, intestinal morphology, and antioxidant capacity. The results showed that the CF group had significantly higher body weight at the end of the nursery period (*p* < 0.05) and a significantly lower post-weaning observed fecal staining rate (*p* < 0.05). At 16–17 days post-weaning, piglets in the CF group exhibited a significant increase in feeding behavior (*p* < 0.05). Compared with the NCF group, the CF group showed highly significant reductions in serum levels of interleukin-6 (IL-6), tumor necrosis factor-alpha (TNF-α), and interferon-gamma (IFN-γ) (*p* < 0.01), as well as significantly increased intestinal superoxide dismutase (SOD) and glutathione peroxidase (GSH-Px) activities (*p* < 0.05) and significantly reduced malondialdehyde (MDA) content (*p* < 0.05). In terms of intestinal morphology, the CF group had a highly significant increase in the villus-to-crypt ratio in the jejunum (*p* < 0.01) and a highly significant reduction in crypt depth (*p* < 0.05), while villus length did not differ significantly between groups (*p* > 0.05). Overall, in the present study, sow–piglet co-feeding during the suckling period effectively alleviated weaning stress and reduced the incidence of diarrhea. These beneficial effects appear to be associated with reduced inflammatory responses, enhanced antioxidant capacity, and improved intestinal morphology. It should be noted that the relatively late weaning age used in this study likely facilitated the piglets’ ability to efficiently utilize solid feed and derive benefits from the co-feeding strategy. Therefore, caution should be exercised when extrapolating these findings to earlier weaning ages, at which the digestive tract is less mature.

## Simple Summary

In modern intensive pig production systems, piglets often experience reduced feed intake, slow body weight gain, and even diarrhea after weaning due to dietary transition stress. This study investigated the effects of a sow–piglet co-feeding strategy during the suckling period, in which piglets had the opportunity to participate in the sow’s feeding process and consumed both lactating sow feed and creep feed. The results showed that, compared with piglets fed only creep feed during lactation, piglets in the co-feeding group had significantly higher body weight at the end of the nursery period, a markedly lower incidence of diarrhea after weaning, and more active feeding behavior on days 16–17 post-weaning. In addition, serum levels of pro-inflammatory cytokines (IL-6, TNF-α, and IFN-γ) were significantly reduced in the co-feeding group. Jejunal antioxidant enzyme activities (SOD and GSH-Px) were enhanced, whereas the content of the oxidative damage marker MDA decreased. Moreover, the villus height-to-crypt depth ratio in the jejunum increased significantly, accompanied by a significant reduction in crypt depth. These findings were obtained in piglets weaned at 36 days of age, a relatively late weaning age compared with common commercial practice. The results suggest that, under these conditions, sow–piglet co-feeding can help alleviate certain aspects of weaning stress and improve intestinal health and production performance. Whether similar benefits can be achieved in piglets weaned at younger ages—whose digestive systems are less mature and less able to utilize solid feed—requires further investigation.

## 1. Introduction

Weaning is a critical event in pig production that can compromise piglet welfare. The abrupt separation from the sow, combined with dietary, social, and environmental changes, readily induces weaning stress, which can severely affect the health and welfare status of piglets [1,2]. Although commercial weaning is often practiced at 3–4 weeks of age, even piglets weaned at a relatively older age (e.g., 5 weeks) still exhibit marked stress responses, including reduced feed intake, intestinal dysfunction, and growth retardation during the immediate post-weaning period. Factors contributing to reduced welfare in weaned piglets include, among others, mixing of litters, changes in housing conditions, nutrition, health status, and aggressive interactions [3,4]. Extending the lactation period can, in some respects, benefit piglet health and welfare by allowing more time for immune maturation and behavioral development [5]. However, prolonged lactation is not without risks, as it may also increase the opportunity for vertical disease transmission from sow to piglet (e.g., Actinobacillus pleuropneumoniae) and is often economically impractical in commercial pig production. Therefore, rather than relying solely on extended lactation, alternative management strategies are needed to improve piglet welfare around weaning.

Although extending the lactation period can mitigate these negative impacts [5], prolonged lactation is often economically impractical in commercial pig production, prompting interest in alternative management strategies such as providing creep feed during the suckling period. Creep feeding aims to familiarize piglets with solid feed before weaning [6,7] and is needed to improve piglet welfare and facilitate the weaning transition, even at the relatively later weaning age of 5 weeks used in the present study. It should be noted, however, that creep feeding is not universally associated with improved welfare; in certain production systems (e.g., outdoor farming), the provision of creep feed may inadvertently attract rodents or increase the risk of pathogen introduction (e.g., *Salmonella* spp.), thereby potentially compromising rather than enhancing welfare. In conventional indoor production systems, where biosecurity and hygiene can be more tightly controlled, creep feeding remains a widely adopted strategy to facilitate the weaning transition.

Creep feeding may also benefit piglet gut health, with studies reporting reductions in lactic acid and acetic acid concentrations in gastric contents, the promotion of gastrointestinal maturation [8], and modulation of the hindgut microbial community structure, which could improve intestinal health [6]. It has been suggested to facilitate intestinal maturation, as reflected by improved crypt depth and villus height, and may enhance barrier function and help mitigate weaning stress-induced intestinal damage [9,10].

Furthermore, creep feeding might alleviate oxidative stress in piglets, potentially by enhancing the activities of glutathione peroxidase (GPx) and glutathione S-transferase (GST), thereby aiding in the removal of free radicals [11]. It may also help reduce intestinal oxidative stress indirectly by improving gut microbiota balance and suppressing the proliferation of pro-oxidative bacterial populations [12,13]. Studies have indicated that, in acute oxidative stress models, piglets receiving creep feed exhibit milder intestinal pathological damage [14,15].

In terms of immune function, creep feed containing lactic acid bacteria (LSB) has been shown to significantly increase total serum IgG levels in piglets [2]. Blood parameter monitoring suggests that creep feeding could help alleviate the stress response associated with weaning [16]. Specific studies examining gene expression related to microbial signaling, barrier function, and immunity indicate that creep feed may influence immune development through the regulation of these genes [8].

However, it is important to recognize that creep feeding does not alleviate all types of weaning stress, nor is its outcome invariably positive. Its benefits are contingent upon multiple factors, including feed composition, intake levels, and overall management conditions. For example, creep feed intake during lactation is often low and highly variable among littermates [17], which can limit its effectiveness. Moreover, if not properly managed, the provision of creep feed can introduce hygiene challenges (e.g., feed spoilage, attraction of pests) that may paradoxically compromise piglet health. Stress reduction, therefore, is not the sole or primary justification for creep feeding; rather, creep feed should be viewed as one component of a comprehensive weaning management program that, when appropriately implemented, can offer meaningful but selective benefits.

Research by Sands et al. [6] comparing different types of creep feed on piglet behavior and growth performance found little negative impact on piglets’ growth performance from consuming sow feed during the nursing period compared to regular creep feed, possibly even better in some aspects. In practical production settings, piglets housed with the sow may naturally participate in the sow’s feeding process, gaining access to the lactating sow diet in addition to creep feed. This shared feeding environment exposes piglets to a mixed diet and introduces social feeding cues from the sow. While many studies have established the benefits of creep feed provision during lactation and the post-weaning phase, the specific effects of sow–piglet co-feeding—where piglets actively share the feeding space and consume both sow feed and creep feed—have received limited attention.

It is important to distinguish sow–piglet co-feeding from the simple provision of creep feed alone. Conventional creep feeding primarily serves to familiarize piglets with solid feed and supply supplemental nutrients before weaning [6,7]. In contrast, co-feeding during the suckling period is a more complex management practice in which piglets not only have access to the sow’s lactating diet in addition to creep feed, but also actively share the feeding space with the sow during her feeding events. This practice introduces several unique components beyond dietary supplementation: (1) piglets consume a mixed diet that is coarser and higher in fiber, providing distinct physical and chemical stimulation to the gastrointestinal tract; (2) the shared feeding environment allows piglets to observe and potentially learn from the sow’s feeding behavior, which may serve as a form of social facilitation and promote earlier and more persistent exploratory and ingestive behaviors; and (3) the co-feeding setup enables piglets to engage in feeding activities in synchrony with the sow, which may help establish more natural feeding rhythms and reduce neophobia toward solid feed. These behavioral and social dimensions, rather than the mere provision of a second feed type, constitute the principal novelty of the sow–piglet co-feeding strategy. Despite its potential practical relevance, the specific effects of this co-feeding approach on post-weaning adaptation, gut health, and overall welfare have received limited research attention.

Research is limited on the practice of sow–piglet co-feeding, and its impact on production performance and healthy farming merits further exploration. Therefore, we hypothesize that a sow–piglet co-feeding strategy during the suckling period—allowing piglets to participate in the sow’s feeding process and consume a mixed diet comprising lactating sow feed and creep feed—can promote the development of exploratory behaviors, increase feed intake during the early post-weaning period, improve growth performance after weaning, alleviate the adverse effects of weaning stress, and enhance intestinal function. This study aims to systematically evaluate the effects of sow–piglet co-feeding on post-weaning growth performance, diarrhea incidence, feeding behavior, intestinal morphology, serum and intestinal immune parameters (including IgA, IgG, SIgA, and inflammatory cytokines such as IL-6, TNF-α, and IFN-γ), as well as jejunal antioxidant capacity (including T-AOC, SOD, GSH-Px, CAT, and MDA). The results of this study are expected to contribute to a growing body of knowledge on pre-weaning nutritional and management interventions, specifically regarding how sow–piglet co-feeding may influence selected aspects of post-weaning adaptation. By focusing on growth performance, diarrhea, feeding behavior, intestinal morphology, immune parameters, and antioxidant capacity, this work aims to provide targeted insights that can inform the development of practical strategies for improving piglet health during the weaning transition.

## 2. Materials and Methods

### 2.1. Animals and Housing

**Figure 1:**
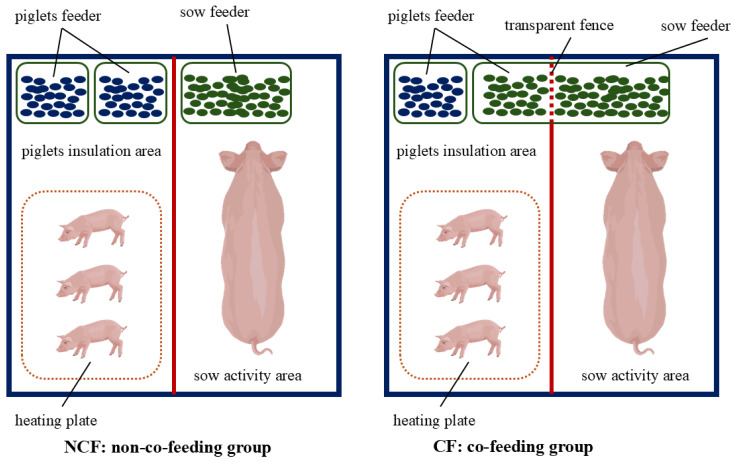
In the NCF group, two piglet feeders of identical size were placed in the piglet insulation area, and the piglets could consume only creep feed. The sow feeders were positioned 25 cm above the ground to physically prevent piglets from accessing the sow feed directly. In the CF group, the piglet insulation area contained one piglet feeder and a second, specially designed feeder connected to the sow feeder. The two feeders were separated by a transparent barrier, allowing the piglets to see the feed in the sow’s trough and to observe the sow’s feeding behavior. Piglets in this group could thus consume both creep feed and lactating sow feed while being exposed to visual social feeding cues from the sow. In both groups, the piglet feeders were placed 1 cm above the ground.

The study was conducted at the A-cheng Experimental Demonstration Base of Northeast Agricultural University in Harbin City, China. The experiment was conducted on 102 newborn piglets (Large White × Duroc × Min Pig). The maternal sows (Duroc × Min Pig) were selected from two distinct genetic lines with well-documented pedigrees, ensuring that genetic background effects could be minimized during statistical analysis. The breeding boar used was an American Large White, with all semen sourced from the same individual. The trial was divided into two treatments: Co-feeding group (*n* = 51): piglets in this group could consume both creep feed and lactating sow feed and could simultaneously participate in the sow’s feeding process. Non-co-feeding group (*n* = 51): piglets had access only to creep feed for suckling piglets. Each treatment consisted of six replicates. The piglet warming area in the pen (1.6 m × 0.8 m) was located at the front side of the sow activity area (4.4 m × 1.6 m), allowing piglets free movement between the warming area and the sow activity area. Seven days prior to farrowing, sows were transferred to farrowing pens to acclimate to the pen environment and the location of the sow feeder. The sow feeder was a standard black, opaque feeder (38 cm length × 36 cm width × 20 cm depth). In the NCF group, the sow feeders were positioned 25 cm above the ground, which physically prevented the piglets from accessing the sow’s feed. In the CF group, a specially designed feeder connected to the sow feeder allowed the transfer of sow feed into the piglet area, while a transparent barrier provided visual access to the sow’s feeding behavior, constituting the sow–piglet co-feeding condition (Figure 1). During the suckling period, each litter was housed with its sow in an individual farrowing pen. After weaning at 36 days of age, piglets were moved to nursery pens where each litter was maintained as an intact social group (i.e., one pen per litter). The facility was well-ventilated, with average temperature and humidity recorded as follows: May—25.22 °C, 62.45%; June—25.93 °C, 72.91%; July—26.83 °C, 76.42%; August—27.98 °C, 79.42%; September—24.63 °C, 61.47%. The pen floor was made of concrete, with daily cleaning and regular disinfection to maintain hygiene. Tooth clipping, castration, and routine husbandry practices are carried out in accordance with the livestock management regulations of the A-cheng Experimental Demonstration Base of Northeast Agricultural University. Both sows and piglets had unrestricted access to clean water throughout the experiment.

In total, the study used 102 piglets from 12 litters (6 litters per treatment, 8–9 piglets per litter on average). Individual body weight measurements were collected from all 102 piglets. Feed intake, fecal staining scores, and behavioral observations were recorded at the litter level during suckling and at the pen level during nursery. A subset of 12 piglets (one piglet per litter, with equal numbers of males and females per treatment) was selected for blood and tissue sampling at 40 days of age.

### 2.2. Feeding Management

After birth, each piglet was individually weighed and tagged. Piglets were allowed to consume colostrum promptly and received standard iron supplementation and immunization procedures; tail docking was not performed. On day 7 after birth, two identical round feeders (R = 12.5 cm, each with four feeding positions) were placed in the piglet warming area, spaced 15–20 cm apart to prevent mixing of spilled feed. In the NCF group, neither feeder contained lactating sow feed; both were filled with equal amounts of creep feed. In the CF group, one of the two feeders contained creep feed, while the other contained an equal amount of lactating sow feed. Before 6:00 each day, piglets were provided with appropriate amounts of creep feed and lactating sow feed for ad libitum consumption. Sows were fed according to the principle of small, frequent meals after farrowing, with feed amounts gradually increased as the sow recovered to ensure adequate milk production. Sow feeding times were 6:00 in the morning, 11:00 at noon, and 17:00 in the afternoon. The nutrient composition and content of feed used in the experiment is shown in Table 1.

At 36 days of age, piglets were weaned at 8:00, weighed individually, and recorded. The grouping of weaned piglets was consistent with the lactation period. After weaning, piglets were reared in the nursery as litter units, with each piglet marked using different colored markers. During the first six days post-weaning, feed was transitioned from creep feed to weaning piglet feed: Day 1—100% creep feed; Day 2—creep feed to weaning feed ratio of 4:1; Day 3—3:2; Day 4—2:3; Day 5—1:4; Day 6—100% weaning feed. Feed was provided daily at 6:00 to ensure ad libitum intake. The remaining feed was weighed the next day, and an appropriate amount was replenished to prevent hunger. Pens were cleaned daily, disinfected regularly, and maintained in hygienic conditions, with unrestricted access to drinking water. Piglet health was monitored daily, and routine nursery management practices were followed. Nursery rearing lasted for 30 days post-weaning, after which piglets were individually weighed and recorded at 8:00 the following day.

The weaning age of 36 days was selected for the following reasons: first, the piglets used in this study were three-way crossbreds (Large White × Duroc × Min Pig) that included a Chinese indigenous breed, which exhibits slower early growth rates compared with commercial lean-type lines; weaning at approximately 5 weeks of age is a common practice for such genotypes in regional production systems. Second, weaning at 36 days allows piglets to reach adequate maturity for solid feed intake while still capturing the post-weaning stress response, providing a suitable window for evaluating the carry-over effects of the suckling feeding strategy.

### 2.3. Growth Performance

Body weight measurements (birth weight, weaning weight, and end-of-nursery weight) were recorded individually for each piglet, and average daily weight gain (ADWG) during the lactation and nursery periods was calculated on an individual piglet basis. In contrast, feed intake data were collected on a litter basis during the suckling period and on a pen basis during the nursery period. Daily feed provision and residual feed (including spilled feed) were recorded per feeder to determine total feed disappearance per litter or pen, and average feed intake per piglet was estimated by dividing total disappearance by the number of piglets in that unit. It should be noted that this provides only a group-level average, not individual feed intake records, and therefore these data were analyzed using litter or pen as the experimental unit. Feed Conversion Ratio (FCR) during the nursery period was calculated at the pen level as total pen feed intake divided by total pen body weight gain over the same period. Consequently, F:G was analyzed statistically using pen as the experimental unit (*n* = 6 per treatment), consistent with the unit at which feed intake was measured.

In the CF group, total feed intake during the suckling period included both creep feed and lactating sow feed consumed from the two feeders. Because piglets were group-housed and had access to both feed types simultaneously, it was not possible to determine the proportion of total intake contributed by each feed type. Therefore, only total feed disappearance per litter is reported, and any inferences about differential consumption of the two diets must remain speculative.

From day 7 to day 65 of age, piglets were observed daily at 16:30 for signs of diarrhea, defined as the presence of yellow, gray, or white fecal matter adhered around the anus. The observed fecal staining rate was recorded separately for the lactation period, the first 1–4 days post-weaning, and the nursery period.

$$\mathrm{Observed}\;\mathrm{fecal}\;\mathrm{staining}\;\mathrm{rate}\;(\%)=\frac{Number\;of\;piglets\;with\;diarrhoea\;during\;the\;experimental\;period}{Number\;of\;piglets\;\times \;Experimental\;days}\;\times \;100\%$$

### 2.4. Behavioral Observation

Behavioral observations of 12 litters of piglets were continuously recorded using the DS-IT5 digital video surveillance system (Hikvision, Hangzhou, China), which consists of high-definition infrared cameras connected to a network video recorder (NVR) for continuous 24 h video capture. All video footage was stored on hard drives and subsequently exported for manual Behavioral scoring by a trained observer. For suckling piglets, continuous video recording was conducted over three consecutive days (Days 7, 8, and 9) following feed provision. Additional recordings were performed on Days 15–16, 22–23, and 29–30 during three specific time periods each day: 06:00–08:00, 11:00–13:00, and 17:00–19:00. To avoid interfering with piglets’ feeding and sleeping periods, and to ensure that no caretakers enter the pens or interact with the piglets during Behavioral observation. A scan sampling method was employed, with observations made at one-minute intervals. Each interval consisted of 10 s of continuous viewing for all piglets, and occurrences of the target behaviors were recorded as a single event; if the behavior was absent, no record was made. The frequency of behavior occurrence within the observation period was used as the measurement index.

For weaned piglets, continuous recording was conducted over the first three days post-weaning (Days 1, 2, and 3). Additional intermittent recording sessions were performed on Days 9–10, 16–17, and 23–24, during the time periods of 09:00–11:00 and 14:00–16:00. Scan sampling was conducted at three-minute intervals, with 10 s of continuous viewing per interval for all piglets. The same recording rules were applied: the occurrence of a target behavior was marked as one event, and absence was not recorded. Behavioral data collection was carried out by a single observer to eliminate inter-observer variability and reduce human error.

### 2.5. Sample Collection

On the fifth day after weaning (at 40 days of age), one piglet was selected from each litter for weighing and subsequent slaughter. To ensure that the selected piglet was representative of the litter, the individual whose body weight was closest to the mean body weight of that litter was chosen. When more than one piglet met this criterion, one was randomly selected. To minimize potential bias, the experimenter performing the selection was blinded to the treatment assignment of the litters. In addition, the selection ensured that each treatment group contained equal numbers of male and female piglets (three of each). Following slaughter, the jejunum was isolated, and a midsection was placed in an ice box containing ice packs. The outer wall and contents were rinsed with pre-chilled physiological saline, blotted dry with filter paper, and the intestinal lumen was opened. The mucosa was scraped onto glass slides, transferred into cryogenic vials, rapidly frozen in liquid nitrogen, and stored at −80 °C. Additionally, a 1 cm intestinal ring segment from the midsection of the jejunum was excised and fixed in 4% paraformaldehyde solution. Prior to slaughter, 5 mL of blood was collected from the anterior vena cava, centrifuged at 3000 rpm for 5 min, and the upper serum layer was aspirated into 1.5 mL EP tubes for storage at −80 °C.

Before blood and tissue collection, piglets were humanely euthanized using an approved electrical stunning device.(Lei Li Technology, Shenzhen, China) The procedure involved the application of sufficient electric current (parameters: 220 V, 50 Hz), for a minimum duration of 5 s across the head to induce immediate loss of consciousness and insensibility. Death was confirmed by the absence of corneal and pedal reflexes before exsanguination and subsequent sampling commenced. This method is consistent with standard protocols for humane slaughter and was reviewed and approved by the Animal Protection and Utilization Committee of Northeast Agricultural University (protocol number NEAUEC20200346).

### 2.6. Histological Examination

The jejunal tissue fixed with 4% paraformaldehyde was dehydrated with ethanol, cleared, and embedded in paraffin to prepare paraffin blocks. Sections of 5 μm thickness were cut and stained with hematoxylin and eosin (HE), followed by imaging using an Eclipse Ci-L upright bright-field microscope (Nikon, Tokyo, Japan). The morphology of the intestinal mucosa was examined with Image-Pro Plus (version 6.0; Media Cybernetics, Inc., Rockville, MD, USA) software, measuring villus length and crypt depth, and calculating the villus-to-crypt ratio. For each piglet, five fields of view were selected from intestinal tissue sections for observation. Villus height was defined as the vertical distance from the tip of the villus to the opening of the crypt, while crypt depth was defined as the vertical distance from the opening of the crypt to its base.

**Figure 2:**
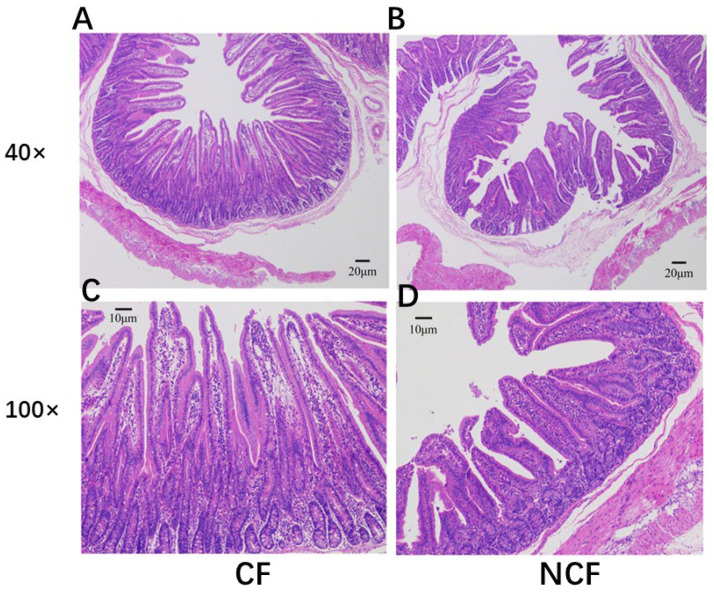
Jejunum morphology. (**A**): Morphology of jejunum in piglets of CF group (40×). (**B**): Morphology of jejunum in piglets of NCF group (40×). (**C**): Morphology of jejunum in piglets of CF group (100×). (**D**): Morphology of jejunum in piglets of NCF group (100×), Scale bars: 20 μm (**A**,**B**); 10 μm (**C**,**D**).

For each piglet, five non-overlapping fields of view were captured from well-oriented sections where the villus structure was clearly visible from tip to base. The images presented in Figure 2 were selected by an observer blinded to the treatment groups, based on the following criteria: the selected image best represented the mean villus height and crypt depth values of the respective experimental group, and was free of sectioning artifacts (such as folds, tears, or uneven staining) that could compromise morphological assessment. This approach ensures that the representative images accurately reflect the group-level morphological characteristics reported in this paper.

### 2.7. Determination of Immunological Indicators

The concentrations of IgA, IgG, IL-1β, IL-4, IL-6, IL-10, IFN-γ, and TNF-α in the serum of each piglet were determined using ELISA kits (Jinma, Shanghai, China), following the manufacturer’s instructions precisely. A suitable amount of jejunal mucosal tissue was mixed with pre-cooled physiological saline at a ratio of 1:9, homogenized using a high-speed tissue grinder (LICHEN, Changsha, China), and centrifuged at 3000 r/min for 10 min. The supernatant was collected for further analysis. The concentration of SIgA in the jejunal mucosa was measured using an SIgA kit (Jinma, Shanghai, China), strictly adhering to the manufacturer’s protocol. SIgA content was normalized to the total protein concentration, which was determined using a BCA protein assay kit (Jinma, Shanghai, China), following the instructions provided by the manufacturer.

### 2.8. Oxidative Stress Indices Measurement

An appropriate amount of jejunal mucosal tissue was mixed with pre-cooled physiological saline at a ratio of 1:9, then homogenized using a high-speed tissue grinder. The homogenate was centrifuged at 3000 r/min for 10 min, and the supernatant was collected for subsequent analysis. The total antioxidant capacity (T-AOC), enzymatic activities of glutathione peroxidase (GSH-Px), catalase (CAT), and superoxide dismutase (SOD), as well as the malondialdehyde (MDA) content in the jejunal mucosa, were determined strictly following the protocols provided in the respective commercial assay kits (Jinma, Shanghai, China). All measurements were normalized to total protein content, which was quantified using a BCA protein assay kit (Jinma, Shanghai, China) in accordance with the manufacturer’s instructions.

### 2.9. Statistical Analysis

All experimental data were initially compiled using Excel 2016 and subsequently analyzed with SPSS 26.0. The piglet data were first subjected to normality testing. Data that did not conform to a normal distribution were transformed using either square root (Sqrt) or natural logarithm (Ln) transformations to achieve normality, followed by tests for homogeneity of variance. For data failing to meet the homogeneity assumption, appropriate transformations were applied to ensure compliance. An independent samples *t*-test based on mean comparisons was employed, using the consumption of co-feeding (CF group vs. NCF group) as the factor, to analyze the effects of different feeding strategies on the growth performance, behavior, immune status, intestinal morphology, and jejunal antioxidant capacity of weaned piglets. For datasets that remained non-normal and heteroscedastic after transformation, the Kruskal–Wallis test under non-parametric procedures was employed. Results are presented as mean ± standard error of the mean (SEM), with *p* < 0.05 considered statistically significant and *p* < 0.01 considered highly significant.

For individually collected data (e.g., body weight, ADWG), the experimental unit was the individual piglet. For group-level data (e.g., ADFI, Feed Conversion Ratio (FCR)), the experimental unit was the litter during suckling or the pen during the nursery period (*n* = 6 per treatment). This distinction was taken into account when applying statistical tests, with group-level data analyzed using pen means.

## 3. Results

### 3.1. Growth Performance

The effects of different feeding methods during the lactation period on piglet growth performance are presented in Table 2 and Table 3. There were no significant differences (*p* > 0.05) in weaning weight, average daily weight gain, or feed intake between piglets in the CF group and those in the NCF group during lactation. However, the body weight at the end of the nursery phase was significantly higher in weaned piglets from the CF group compared with those from the NCF group (*p* < 0.05). No significant differences (*p* > 0.05) were observed between the two groups in average daily weight gain, average daily feed intake, Feed Conversion Ratio (FCR), or average daily feed intake during days 1–4 post-weaning.

### 3.2. Diarrhea

As shown in Table 4, there was no significant difference in diarrhea incidence between suckling piglets in the CF group and those in the NCF group (*p* > 0.05). However, the observed fecal staining rate of weaned piglets in the CF group was significantly lower than that of the NCF group (*p* < 0.05). During the nursery period, no significant difference in diarrhea incidence was observed between the CF and NCF groups within 1–4 days post-weaning (*p* > 0.05).

### 3.3. Behavior

The behavioral outcomes of the two groups are presented in Table 5. For most of the observation period, piglets in the CF group exhibited no significant differences (*p* > 0.05) from those in the NCF group in either feed intake behavior or feed spillage intake behavior. Interestingly, however, at 51–52 days of age (the 16th–17th day post-weaning), the CF group demonstrated significantly higher frequencies of both feed intake behavior and feed spillage intake behavior compared with the NCF group (*p* < 0.05).

### 3.4. Immunological Indicators

Table 6 presents the effects of different feeding methods during lactation on the immune status of weaned piglets. No significant differences (*p* > 0.05) were observed between the CF group and the NCF group in terms of serum IgA, IgG, IL-1β, IL-4, IL-10, and intestinal mucosal SIgA levels. However, the concentrations of IL-6, TNF-α, and IFN-γ in the CF group were significantly lower than those in the NCF group (*p* < 0.01).

### 3.5. Oxidative Stress Indexes

Table 7 presents the effects of different feeding methods during lactation on the antioxidant capacity of the jejunum in weaned piglets. The total antioxidant capacity (T-AOC) and catalase (CAT) activity in the jejunum of piglets in the CF group did not differ significantly from those in the NCF group (*p* > 0.05). However, superoxide dismutase (SOD) activity was significantly higher in the CF group than in the NCF group (*p* < 0.05), and glutathione peroxidase (GSH-Px) activity was markedly higher (*p* < 0.01). In contrast, the malondialdehyde (MDA) content was significantly lower in the CF group compared with the NCF group (*p* < 0.05).

### 3.6. Jejunal Morphology

Table 8 and Figure 2 presents the effects of different feeding methods during lactation on the jejunal morphology of weaned piglets. The jejunal villus length in the CF group did not differ significantly from that in the NCF group (*p* > 0.05). However, the crypt depth was significantly lower in the CF group compared with the NCF group (*p* < 0.05), and the villus-to-crypt ratio was markedly higher in the CF group than in the NCF group (*p* < 0.01).

## 4. Discussion

### 4.1. Growth Performance

This study demonstrates that feeding lactating piglets with either creep feed or a co-feeding (NCF group vs. CF group) during the lactation period resulted in no statistically significant differences in growth performance. Sands et al. [6], who compared different types of creep feed during lactation, also reported no statistically significant differences in growth performance, a finding that aligns with the absence of detectable differences observed under the present experimental conditions. It should be noted, however, that failure to detect a significant difference does not confirm equivalence or the absence of an effect; it may partly reflect limited statistical power at the group level (*n* = 6 litters per treatment) to detect smaller but biologically meaningful differences. These results suggest that under the conditions of this study, allowing piglets access to sow lactation feed during the suckling period did not impair their growth performance during lactation. However, the current trial was not designed to test for equivalence; therefore, the data do not exclude the possibility of modest positive or negative effects that fell below the detection threshold. Although the differences in weaning weight and average daily weight gain during lactation did not reach statistical significance under the current trial conditions, piglets in the CF group were numerically 2.23 kg heavier at weaning (10.05 ± 0.66 kg) than those in the NCF group (7.82 ± 0.67 kg), and ADWG was numerically 36.84% higher. These numerically higher values are noteworthy and raise the possibility of a biologically relevant effect that would require a larger study to confirm or refute.

The weaning piglets in the CF group had significantly higher body weights at the end of the nursery period compared with those in the NCF group. However, no significant differences were observed between the two groups in average daily weight gain, average daily feed intake, feed intake during days 1–4 post-weaning, or Feed Conversion Ratio (FCR). Sands et al. [6] reported no statistically significant differences in these nursery-period outcomes under their experimental conditions, which involved mixing of piglets after weaning and a different genetic background. The significant difference in nursery-end body weight observed in the present study, but not in Sands et al. [6], may therefore reflect differences in weaning age, social stress associated with mixing, or genetic factors. These discrepancies underscore the context-dependent nature of the response to pre-weaning feeding strategies. The discrepancy may be attributed to the mixing of weaned piglets in Sands et al.’s study, which can impose substantial social stress, leading to fighting [7,8,9]. Increased fighting time reduces feed intake, and the resulting skin injuries can cause inflammation, immunosuppression, and growth retardation [10], thereby affecting both growth performance and welfare. Although the differences in nursery-end body weight and average daily weight gain were not statistically significant in the present study, CF group piglets had numerically higher nursery-end body weight (25.51 ± 1.27 kg vs. 19.66 ± 1.50 kg) and average daily weight gain (0.51 ± 0.03 kg/d vs. 0.39 ± 0.04 kg/d), as well as a lower Feed Conversion Ratio (FCR) (1.54 ± 0.07 vs. 1.80 ± 0.12) compared with NCF group piglets, suggesting a performance advantage associated with co-feedinging.

It should be noted that the weaning age employed in this study (36 days) is later than that practiced in many intensive commercial systems where piglets are commonly weaned at 21–28 days. Older weaning age is associated with greater solid feed intake during lactation and a more mature digestive system at weaning, which may partially attenuate the severity of weaning stress and enhance the piglet’s capacity to benefit from pre-weaning feeding interventions [5,10]. Therefore, the beneficial effects of sow–piglet co-feeding observed here may not be directly extrapolated to earlier weaning scenarios, and further investigation is warranted to determine whether similar benefits can be achieved at younger weaning ages. Additionally, it should be acknowledged that the between-litter coefficient of variation for weaning weight exceeded 20% in both treatment groups. This substantial variation likely reflects, in addition to inherent biological differences among litters, individual differences in creep feed and sow feed intake during the suckling period, which is known to be highly variable, with some individuals consuming negligible amounts while others consume considerably more [17]. In the CF group, this variation may have been further influenced by the extent to which individual piglets participated in the co-feeding process and their competitive success in accessing the sow feeder. These sources of variation, combined with the relatively small number of experimental units at the litter level, reduce the statistical power to detect treatment effects on weaning weight. This limitation should be considered when interpreting the non-significant *p* value for weaning weight (*p* = 0.064) despite the large numerical difference between groups.

### 4.2. Diarrhea

The incidence of diarrhea in suckling piglets from the CF group did not differ significantly from that in the NCF group; however, it was reduced by 46.85%, which holds considerable economic value in practical production. This reduction in diarrhea may be primarily attributed to the higher fiber content in the lactating sow feed consumed by piglets. Intake of higher-fiber diets by piglets has been reported to lower observed fecal staining rates [10,11]. In this study, the fiber content of the lactating sow diet was 7%. Previous research has indicated that a fiber content of 5.3–5.5% in piglet diets may effectively reduce diarrhea [12]. The fiber content in the suckling piglet feed in this study was 4%. Based on the average fiber content of the two diets, the actual fiber intake of piglets consuming the co-feeding was approximately 5.5%, which is close to the optimal range and conducive to reducing diarrhea.

Post-weaning diarrhea is a major cause of poor growth performance during the nursery period [13]. The present study demonstrated that the observed fecal staining rate in weaned piglets from the CF group was significantly lower than that in the NCF group throughout the nursery stage. This result may be related to the co-feeding treatment as a whole, which combined access to lactating sow feed with exposure to the sow’s feeding behavior and the associated social cues. The co-feeding experience during lactation—which included exposure to higher-fiber sow feed—may have facilitated adaptation to the weaning diet and contributed to reduced stress levels [14]. The co-feeding strategy, through the provision of sow feed with a higher fiber content, may also have promoted superior intestinal development, thereby reducing gastrointestinal burden after weaning [15]. It is possible that the ingestion of sow feed during lactation, which contains higher fiber, could promote the proliferation of fiber-degrading microorganisms, thereby potentially facilitating a more rapid adaptation of the gut microbiota after weaning and contributing to the reduced diarrhea incidence observed [16]. However, this hypothesis remains to be tested through dedicated microbiota analysis in future studies.

It should be acknowledged that the fecal staining rate recorded in this study is a proxy indicator and likely underestimates the true incidence of diarrhea. The single daily observation at a fixed time point may miss diarrheic episodes occurring at other times or cases in which the perianal area was cleaned by the sow or littermates. Conversely, it is also possible that some instances of fecal staining were not associated with clinical diarrhea. Therefore, the observed differences between groups should be interpreted as a conservative indication of improved digestive comfort in the CF group rather than a precise quantification of clinical disease incidence.

### 4.3. Behavior

Under the conditions of the present study, no statistically significant differences in feeding behavior were detected between suckling piglets in the NCF and CF groups. This observation is similar to that of Clouard et al. [17], who also reported no significant behavioral differences when providing fibrous diets to suckling piglets. It should be emphasized that the absence of statistically significant differences does not confirm the absence of an effect; rather, it indicates that any potential effect was not large enough to be detected with the current sample size (*n* = 6 litters per treatment) and within the specific feed formulations and feeder design tested. Different feed formulas or feeder arrangements could potentially yield different outcomes.

The possible reasons for this outcome may include that a high-fiber diet does not affect foraging-related activities in suckling piglets; that feed with elevated fiber content has little or no impact on energy intake; or that any nutritional differences caused by consuming sow feed with higher fiber levels could be compensated for by milk intake. However, the precise mechanisms underlying these results require further investigation.

In the present study, during the early post-weaning period (days 1–10 after weaning), no statistically significant differences in feeding behavior were observed between the CF and NCF groups. Accordingly, no evidence was found for an effect of the co-feeding treatment on early post-weaning feeding behavior under the current experimental conditions. Whether co-feeding with different feed types or under different management conditions would influence early post-weaning feeding behavior remains an open question. One possible explanation for the absence of detectable behavioral differences in the early post-weaning period is that post-weaning feeding behavior may be more strongly influenced by factors other than pre-weaning diet composition, such as the degree of environmental enrichment or social dynamics within the pen. However, this hypothesis cannot be tested with the current data and requires further investigation. This hypothesis, however, requires further experimental validation. Notably, on days 51–52 (days 16–17 after weaning), piglets in the CF group exhibited significantly higher frequencies of both feed consumption and feed spillage behaviors compared with those in the NCF group, which could be beneficial for growth and development. The likely explanation is that stress-induced damage caused by weaning had begun to recover [18], resulting in increased nutritional demands that drove piglets to consume more feed, thereby increasing the frequency of feeding behaviors.

It is noteworthy that the observed difference in feeding behavior between the CF and NCF groups reached statistical significance only on days 51–52 (days 16–17 post-weaning), but not during the immediate post-weaning period (days 1–10). This temporal pattern suggests that co-feeding during the suckling period may not directly stimulate early feed intake immediately after weaning; instead, it may facilitate the recovery process from weaning stress and promote a more robust resumption of feeding activity once the acute stress phase has subsided. This interpretation is consistent with the notion that stress-induced damage begins to recover during the second and third weeks post-weaning [19,20]. Therefore, the behavioral benefit of co-feeding appears to manifest as a sustained improvement in feeding motivation rather than an immediate alleviation of feed neophobia.

Overall, the behavioral data from this study do not provide evidence for an effect of sow–piglet co-feeding on feeding behavior, either during the suckling period or in the immediate post-weaning phase. However, this finding should be interpreted with caution. It should be noted that several numerical differences in feeding behavior between the CF and NCF groups during both the late suckling and early nursery periods were relatively large in magnitude but did not reach statistical significance (Table 5), largely attributable to the high within-group variability inherent in behavioral data, particularly during lactation when piglet activity is influenced by the sow’s presence, and the relatively limited number of experimental units at the pen level (*n* = 6 per treatment). The study was not designed as an equivalence or non-inferiority trial, and the statistical power to detect behavioral differences was accordingly limited. Furthermore, the observed behavioral outcomes are specific to the feed formulations (4% vs. 7% crude fiber in creep feed and sow feed, respectively) and the transparent-barrier feeder design employed. Although these numerical differences did not achieve conventional statistical thresholds, their direction and consistency across time points suggest a trend toward more active feeding in CF piglets that may have biological relevance. Whether alternative feed formulations, different feeder configurations, or larger group sizes would reveal behavioral effects of co-feeding remains to be investigated. Therefore, the present results do not rule out the possibility that co-feeding influences feeding behavior; they simply indicate that no such influence was detectable under the conditions of this experiment. Future studies with larger sample sizes at the pen level are recommended to increase statistical power for detecting behavioral effects.

### 4.4. Immunological Indicators

In the present study, no statistically significant differences were detected between the CF and NCF groups in serum IgA, IgG, or intestinal mucosal IgA concentrations. This observation does not preclude the possibility that co-feeding modulates humoral immunity under different experimental conditions. The absence of detectable differences may be attributed to several factors, including the specific feed formulations used (4% vs. 7% crude fiber), the relatively late weaning age of 36 days, which may have allowed for sufficient passive immune acquisition, and the limited sample size at the individual piglet level (*n* = 6 per treatment). It is also possible that the measured time point (day 5 post-weaning) was not optimal for capturing transient changes in immunoglobulin levels. Other researchers have reported that dietary fiber supplementation can increase serum IgM, IgG, and intestinal SIgA in weaned piglets [21,22], but those studies typically involved post-weaning fiber provision and different diet compositions. Therefore, the present findings cannot be directly compared to such studies, and the potential immunomodulatory role of sow–piglet co-feeding remains open to investigation with different diet formulas, earlier weaning ages, or larger sample sizes.

Numerous studies have demonstrated that other immune responses occurring during weaning stress involve alterations in cytokine production [23,24]. In this study, serum levels of the pro-inflammatory cytokines IL-6, TNF-α, and IFN-γ were lower in CF piglets compared with NCF piglets, while the anti-inflammatory cytokine IL-10 tended to be higher. These findings are consistent with Neurath [25], who observed that feeding citrus pectin to piglets reduced intestinal IL-6 levels; elevated IL-6 can impair the intestinal epithelial barrier and contribute to inflammatory bowel disease. The present results suggest that consumption of co-feeding during the suckling period can reduce post-weaning inflammation and enhance piglet immunity. Other studies have shown that apple pectin supplementation can lower intestinal IL-6 and TNF-α levels in mice fed high-fat diets [26]. In patients undergoing hemodialysis, supplementation with 10 g or 20 g of dietary fiber significantly increased total antioxidant capacity (T-AOC), reduced malondialdehyde (MDA) levels, and markedly decreased TNF-α, IL-6, IL-8, and C-reactive protein (CRP) concentrations [27]. These findings further support the anti-inflammatory potential of the sow–piglet co-feeding strategy employed during the suckling period.

### 4.5. Oxidative Stress Indexes

After weaning, piglets experience stress that enhances oxidative processes in the body. Total antioxidant capacity (T-AOC) reflects the overall antioxidant potential of the organism [28]. Superoxide dismutase (SOD) can catalyze the dismutation of free radicals, thereby alleviating oxidative stress responses [29]. Malondialdehyde (MDA), a metabolite of lipid peroxidation [22], is widely recognized as an important biomarker of oxidative stress; higher MDA levels indicate stronger oxidative toxicity.

In this study, the CF group of weaned piglets exhibited higher SOD and GSH-Px activities and lower MDA concentrations compared to the NCF group, with T-AOC and CAT activities showing an upward trend relative to the NCF group. The stronger antioxidant capacity observed in CF piglets after weaning may be attributed, on one hand, to the co-feeding treatment, which included access to lactating sow feed, may have enhanced intestinal antioxidative function, and on the other hand, to the lower stress levels and reduced intestinal oxidative damage resulting from the more palatable feed provided post-weaning.

### 4.6. Jejunal Morphology

In addition to impairing immune function and antioxidant capacity, weaning in piglets induces marked alterations in intestinal morphology, such as villus atrophy and crypt deepening [20]. Villus height, crypt depth, and the villus-to-crypt ratio are key morphological indices that can, to some extent, reflect intestinal functionality and health status [30]. Greater villus height corresponds to a larger absorptive surface area, facilitating nutrient uptake; shallower crypts indicate higher epithelial maturity, thereby enhancing nutrient absorption capacity [31]. A higher villus-to-crypt ratio suggests faster epithelial turnover and improved intestinal health. Studies have shown that weaning stress significantly affects the morphological structure of the small intestine, with the most severe impact occurring on the third day after weaning, and normal morphology being restored only by the fourteenth day [19].

In the present study, piglets in the CF group exhibited shallower crypt depth and higher villus-to-crypt ratios compared to the NCF group, with a tendency toward greater villus length. These findings are largely consistent with the results reported by Diao et al. [20], who found that dietary inclusion of a certain proportion of beet pulp increased villus length and villus-to-crypt ratios in the jejunum and ileum of pigs. The superior intestinal morphology observed in CF piglets may be attributed to the co-feeding experience as a whole, which provided a combination of dietary variety and social feeding cues that together constituted a form of intestinal training, thereby improving intestinal structure and promoting intestinal development. Plant-based feeds have been shown to enhance villus length and reduce crypt depth, facilitating nutrient digestion and absorption [32]. Although fiber level was not a controlled treatment factor in the present study, it is worth noting that the sow–piglet co-feeding strategy allowed CF piglets to access a diet with a higher crude fiber content (approximately 7% in the sow feed vs. 4% in the creep feed). While the contribution of this fiber difference to the observed morphological improvements cannot be isolated from other aspects of the co-feeding treatment (e.g., social feeding cues, feeder design), previous studies have reported that increased dietary fiber intake can be associated with increased intestinal mass, greater goblet cell numbers, and improved gut morphology in piglets [33]. These findings provide a plausible mechanistic context for the morphological benefits observed in the CF group, but direct confirmation of the specific role of fiber within the co-feeding paradigm requires further targeted investigation.

### 4.7. Limitations

This study has several limitations. (1) Although the sows used as dams originated from the same two genetic lines with well-defined pedigrees, thereby controlling for potential effects of differing genetic backgrounds, litter allocation was not strictly randomized and may still have introduced potential selection bias. Future studies should employ experimental designs with secure random allocation at the single-litter or individual piglet level to strengthen causal inference. (2) The piglets used in the experiment were three-way crossbreds incorporating the Chinese indigenous breed Northeast Min pig. This breed is known for a rougher body conformation, slower growth, and potentially earlier intestinal maturation compared with the lean commercial breeds widely used in intensive global production systems (e.g., Large White, Landrace, and Pietrain). The inherent early maturity of Min pigs and their potentially greater resistance to digestive disorders may influence the magnitude and generalizability of the co-feeding response. Therefore, the present results are most applicable to regional production systems adopting similar crossbreeding schemes and weaning ages. Extrapolation to commercial systems involving earlier weaning and highly selected lean genotypes should be undertaken with caution. Future multi-site studies using earlier weaning ages and a broader range of commercial breeds are warranted to evaluate the wider applicability of sow–piglet co-feeding as a post-weaning improvement strategy. (3) The diets used in the experiment were commercial products (Wellhope Agri-Tech Co., Ltd., Shenyang, China), and their complete specifications are proprietary information; consequently, detailed nutrient compositions and ingredient formulations of the creep feed and the lactating sow diet cannot be reported. Only guaranteed analyses of basic nutrient categories are available (see Table 1), which limits the ability to identify the contribution of specific dietary components to the observed effects. (4) The experimental design did not include a positive control group in which piglets received the sow diet without exposure to the sow’s feeding behavior. Therefore, it cannot be determined whether the observed benefits resulted from the consumption of the sow diet itself, the social learning opportunity provided by the transparent barrier, or a synergistic interaction between the two. This study aimed to evaluate the overall effectiveness of the sow–piglet co-feeding strategy as an integrated management practice; however, disentangling the relative contributions of dietary factors and social learning in future research will require a 2 × 2 factorial design. (5) Behavioral endpoints were assessed with a limited number of experimental units at the pen level (*n* = 6 per treatment), and after correction for multiple comparisons no behavioral differences reached statistical significance, indicating that the study was underpowered to detect behavioral effects.

## 5. Conclusions

Sow–piglet co-feeding during the suckling period can effectively alleviate weaning stress, reduce the incidence of diarrhea, and achieve these benefits by mitigating inflammation, enhancing antioxidant capacity, and improving intestinal morphology. Further studies are needed to evaluate the efficacy of this strategy in earlier weaning systems and in piglets of typical commercial genotypes.

## Figures and Tables

**Table 1:** Nutrient composition and content of feed.

Nutritional Components	Creep Feed	Sow Feed	Weaned Piglet Feed
	Content (%)		
Crude protein	18.0	17.0	16.5
Coarse fiber	4.0	7.0	6.0
Crude ash	7.0	9.0	7.0
Moisture	14.0	14.0	14.0
Total phosphorus	0.5	0.5	0.45
Sodium chloride	0.3–1.49	0.3–1.2	0.3–1.2
Calcium	0.4–1.5	0.7–1.3	0.5–1.2
Lysine	1.35	1.1	1.2

**Table 2:** Effects of sow–piglet co-feeding on individual piglet growth performance.

Index (Individual Level)	NCF	CF	*p*-Value
Newborn weight (kg)	1.15 ± 0.03	1.16 ± 0.01	0.515
Weaning weight	7.82 ± 0.67	10.05 ± 0.66	0.153
ADWG (suckling period, kg)	0.19 ± 0.02	0.26 ± 0.02	0.144
End-of-nursing weight (kg)	19.66 ± 1.50	25.51 ± 1.27	0.046
ADWG (nursing period, kg)	0.39 ± 0.04	0.51 ± 0.03	0.078

Abbreviations: CF: co-feeding group, NCF: non-co-feeding group; ADWG = Average daily weight gain. Data are presented as mean ± SEM. *n* = 51 piglets per treatment. Statistical analysis used individual piglet as the experimental unit.

**Table 3:** Effects of sow–piglet co-feeding on litter/pen-level feed intake and feed efficiency.

Index (Litter/Pen Level)	NCF	CF	*p*-Value
ADFI (kg)	0.68 ± 0.04	0.78 ± 0.05	0.156
Feed intake (suckling period, g)	413.06 ± 69.41	423.69 ± 46.69	0.962
Feed intake of 36–39 day (kg)	0.34 ± 0.02	0.44 ± 0.03	0.284
Feed Conversion Ratio (FCR, kg feed/kg gain)	1.80 ± 0.12	1.54 ± 0.07	0.343

Abbreviations: CF: co-feeding group, NCF: non-co-feeding group; ADFI = Average daily feed intake, FCR was calculated at the pen level as total pen feed intake (kg) divided by total pen body weight gain (kg) over the nursery period (*n* = 6 pens per treatment). Data are presented as mean ± SEM. *n* = 6 litters/pens per treatment. Feed intake was measured at the litter level during suckling and at the pen level during the nursery period; values per piglet were obtained by dividing total litter/pen intake by the number of piglets in that unit. Statistical analysis used litter or pen as the experimental unit.

**Table 4:** Effects of sow–piglet co-feeding on diarrhea of piglets.

Observed Fecal Staining Rate (%)	NCF	CF	*p*-Value
Suckling period	17.34 ± 1.50	11.84 ± 0.93	0.053
Weaning period	19.42 ± 1.15	13.54 ± 0.88	0.016

Abbreviations: CF: co-feeding group, NCF: non-co-feeding group. Fecal staining rate (%) was calculated on a litter basis during the suckling period and on a pen basis during the weaning period. Data are presented as mean ± SEM; *n* = 6 experimental units (litters or pens) per treatment. Statistical comparisons between CF and NCF groups were performed using an independent samples *t*-test after confirming normality and homogeneity of variance assumptions. For variables that did not meet parametric assumptions after transformation, the Mann–Whitney U test was used.

**Table 5:** Effects of sow–piglet co-feeding on feeding behavior of piglets.

Index (Times/Pig)		NCF	CF	*p*-Value
7–9 day	Eating feed within feeder	11.07 ± 1.65	11.44 ± 1.86	0.968
	Eating spilled feed of feeder	3.85 ± 0.86	5.46 ± 0.76	0.950
15–16 day	Eating feed within feeder	7.88 ± 1.83	10.17 ± 1.03	0.492
	Eating spilled feed of feeder	2.21 ± 0.52	3.42 ± 0.70	0.946
22–23 day	Eating feed within feeder	12.78 ± 3.72	12.30 ± 0.98	0.638
	Eating spilled feed of feeder	2.82 ± 0.63	5.09 ± 1.00	0.739
29–30 day	Eating feed within feeder	12.53 ± 2.82	16.20 ± 1.66	0.666
	Eating spilled feed of feeder	2.94 ± 0.43	4.78 ± 0.85	0.811
36–38 day	Eating feed within feeder	83.13 ± 9.77	99.82 ± 6.90	0.745
	Eating spilled feed of feeder	9.87 ± 2.85	13.83 ± 1.83	0.361
44–45 day	Eating feed within feeder	15.13 ± 2.03	20.73 ± 1.23	0.158
	Eating spilled feed of feeder	4.24 ± 1.13	5.39 ± 1.10	0.340
51–52 day	Eating feed within feeder	14.90 ± 1.75	23.78 ± 1.57	0.047
	Eating spilled feed of feeder	2.99 ± 0.51	4.97 ± 0.31	0.043
58–59 day	Eating feed within feeder	16.91 ± 0.73	20.20 ± 0.76	0.089
	Eating spilled feed of feeder	2.83 ± 1.09	4.07 ± 0.45	0.304

Abbreviations: CF: co-feeding group, NCF: non-co-feeding group. Data are presented as mean ± SEM. *n* = 6 litters per treatment during the suckling period (7–30 days) and *n* = 6 pens per treatment during the post-weaning period (36–59 days). Statistical comparisons between CF and NCF groups were performed at each observation period using an independent samples *t*-test. For variables that remained non-normally distributed after transformation, the Mann–Whitney U test (non-parametric) was applied. Statistical significance was set at *p* < 0.05.

**Table 6:** Effects of sow–piglet co-feeding on immune parameters of piglets.

Index (μg/mL)	NCF	CF	*p*-Value
IgA	39.60 ± 0.56	41.63 ± 0.57	0.308
IgG	486.82 ± 2.27	499.32 ± 2.87	0.112
IL-1β	36.79 ± 1.13	34.45 ± 0.39	0.133
IL-4	102.34 ± 0.33	104.46 ± 0.86	0.688
IL-6	294.37 ± 4.16	238.38 ± 2.90	*p* < 0.001
IL-10	108.17 ± 0.65	109.57 ± 0.45	0.092
TNF-α	411.83 ± 6.05	375.50 ± 5.04	0.006
IFN-γ	417.62 ± 6.19	365.80 ± 8.48	0.009
SIgA	8.34 ± 0.06	8.91 ± 0.16	0.140

Abbreviations: CF: co-feeding group, NCF: non-co-feeding group. Results were expressed as mean ± standard error (Mean ± SEM). Data are presented as mean ± SEM. *n* = 6 piglets per treatment (one piglet per litter; 3 males and 3 females per group). Statistical comparisons between CF and NCF groups were performed using an independent samples *t*-test after confirming normality and homogeneity of variance assumptions. For variables that did not meet parametric assumptions after transformation, the Mann–Whitney U test was used.

**Table 7:** Effects of sow–piglet co-feeding on oxidative stress indices in the piglets.

Index	NCF	CF	*p*-Value
T-AOC (U/mg prot)	0.71 ± 0.02	0.80 ± 0.02	0.189
SOD (U/mg prot)	20.31 ± 0.56	22. 13 ± 0.29	0.034
GSH-Px (U/mg prot)	15.80 ± 0.25	20.28 ± 0.65	*p* < 0.001
CAT (U/mg prot)	2.88 ± 0.04	3.16 ± 0.07	0.294
MDA (nmol/mg prot)	1.24 ± 0.02	1.12 ± 0.02	0.041

Abbreviations: CF: co-feeding group, NCF: non-co-feeding group. Results were expressed as mean ± standard error (Mean ± SEM). Data are presented as mean ± SEM. *n* = 6 piglets per treatment (one piglet per litter; 3 males and 3 females per group). Statistical comparisons between CF and NCF groups were performed using an independent samples *t*-test after confirming normality and homogeneity of variance assumptions. For variables that did not meet parametric assumptions after transformation, the Mann–Whitney U test was used.

**Table 8:** Effects of sow–piglet co-feeding on jejunal morphology of weaned piglets.

Index	NCF	CF	*p*-Value
Villus length (mm)	0.34 ± 0.01	0.44 ± 0.02	0.067
Crypt depth (mm)	0.29 ± 0.01	0.22 ± 0.01	0.011
Villus crypt ratio	1.16 ± 0.07	2.11 ± 0.22	0.008

Abbreviations: CF: co-feeding group, NCF: non-co-feeding group. Results were expressed as mean ± standard error (Mean ± SEM). Data are presented as mean ± SEM. *n* = 6 piglets per treatment (one piglet per litter; 3 males and 3 females per group). Statistical comparisons between CF and NCF groups were performed using an independent samples *t*-test after confirming normality and homogeneity of variance assumptions. For variables that did not meet parametric assumptions after transformation, the Mann–Whitney U test was used.

## Data Availability

The original contributions presented in this study are included in the article. Further inquiries can be directed to the corresponding author(s).

## Abbreviations

The following abbreviations are used in this manuscript:

CF	Co-feeding group
NCF	Non-co-feeding group
IL	Interleukin
TNF	Tumor Necrosis Factor
IFN	Interferon
T-AOC	Total antioxidant capacity
GSH-Px	Glutathione peroxidase
CAT	Catalase
MDA	Malondialdehyde
ADWG	Average daily weight gain
ADFI	Average daily feed intake
FCR	Feed Conversion Ratio
Ig	Immunoglobulin
SOD	Superoxide Dismutase

## References

[1] Tang X.P., Xiong K.N., Fang R.J., Li M.J. (2022). Weaning stress and intestinal health of piglets: A review. Front. Immunol..

[2] Sun H.Q., de Laguna F.B., Wang S., Liu F.J., Shi L., Jiang H.D., Hu X.X., Qin P., Tan J.J. (2022). Effect of *Saccharomyces cerevisiae boulardii* on sows’ farrowing duration and reproductive performance, and weanling piglets’ performance and IgG concentration. J. Anim. Sci. Technol..

[3] O’Doherty J., Dowley A., Conway E., Sweeney T. (2024). Nutritional Strategies to Mitigate Post-Weaning Challenges in Pigs: A Focus on Glucans, Vitamin D, and Selenium. Animals.

[4] Correa F., Luise D., Palladino G., Scicchitano D., Brigidi P., Martelli P.L., Babbi G., Turroni S., Litta G., Candela M. (2023). Influence of body lesion severity on oxidative status and gut microbiota of weaned pigs. Animal.

[5] Kongsted H., Sorensen J.T. (2017). Lesions found at routine meat inspection on finishing pigs are associated with production system. Vet. J..

[6] Sands J.M., Rodrigues L.A., Wellington M.O., Panisson J.C., Columbus D.A. (2022). Pre- and post-weaning performance of piglets offered different types of creep feed. Can. J. Anim. Sci..

[7] Turner S.P., Nevison I.M., Desire S., Camerlink I., Roehe R., Ison S.H., Farish M., Jack M.C., D’Eath R.B. (2017). Aggressive behaviour at regrouping is a poor predictor of chronic aggression in stable social groups. Appl. Anim. Behav. Sci..

[8] Coutellier L., Arnould C., Boissy A., Orgeur P., Prunier A., Veissier I., Meunier-Salaün M.C. (2007). Pig’s responses to repeated social regrouping and relocation during the growing-finishing period. Appl. Anim. Behav. Sci..

[9] Ji W.B., Bi Y.J., Cheng Z., Liu R.Z., Zhang X.H., Shu Y.F., Li X., Bao J., Liu H.G. (2021). Impact of early socialization environment on social behavior, physiology and growth performance of weaned piglets. Appl. Anim. Behav. Sci..

[10] Gerritsen R., van der Aar P., Molist F. (2012). Insoluble nonstarch polysaccharides in diets for weaned piglets. J. Anim. Sci..

[11] Molist F., de Segura A.G., Pérez J.F., Bhandari S.K., Krause D.O., Nyachoti C.M. (2010). Effect of wheat bran on the health and performance of weaned pigs challenged with K88. Livest. Sci..

[12] Yan C.L., Kim H.S., Hong J.S., Lee J.H., Han Y.G., Jin Y.H., Son S.W., Ha S.H., Kim Y.Y. (2017). Effect of Dietary sugar beet pulp supplementation on growth performance, nutrient digestibility, fecal Microflora, blood profiles and Diarrhea incidence in weaning pigs. J. Anim. Sci. Technol..

[13] Heo J.M., Kim J.C., Yoo J., Pluske J.R. (2015). A between-experiment analysis of relationships linking dietary protein intake and post-weaning diarrhea in weanling pigs under conditions of experimental infection with an enterotoxigenic strain of *Escherichia coli*. Anim. Sci. J..

[14] Pu G., Hou L.M., Du T.R., Zhou W.D., Liu C.X., Niu P.P., Wu C.W., Bao W.B., Huang R.H., Li P.H. (2023). Increased Proportion of Fiber-Degrading Microbes and Enhanced Cecum Development Jointly Promote Host to Digest Appropriate High-Fiber Diets. Msystems.

[15] Shang Q.H., Liu H.S., Wu D., Mahfuz S., Piao X.S. (2021). Source of fiber influences growth, immune responses, gut barrier function and microbiota in weaned piglets fed antibiotic-free diets. Anim. Nutr..

[16] Zhang B., Zheng T.H., He Z.T., Su S.L., Yuan S.Y., Chen D.P., Li H.B., Guan W.T., Zhang S.H. (2025). Maternal purified fiber supplementation-enriched *Akkermansia muciniphila* regulates lactation and offspring growth via the gut-mammary axis. Sci. China Life Sci..

[17] Clouard C., Stokvis L., Bolhuis J.E., van Hees H.M.J. (2018). Short communication: Insoluble fibres in supplemental pre-weaning diets affect behaviour of suckling piglets. Animal.

[18] Montagne L., Boudry G., Favier C., Le Huërou-Luron I., Lallès J.P., Sève B. (2007). Main intestinal markers associated with the changes in gut architecture and function in piglets after weaning. Brit J. Nutr..

[19] Wang M., Wang L.X., Tan X., Wang L., Xiong X., Wang Y.C., Wang Q.Y., Yang H.S., Yin Y.L. (2022). The developmental changes in intestinal epithelial cell proliferation, differentiation, and shedding in weaning piglets. Anim. Nutr..

[20] Diao H., Jiao A.R., Yu B., He J., Zheng P., Yu J., Luo Y.H., Luo J.Q., Mao X.B., Chen D.W. (2020). Beet Pulp: An Alternative to Improve the Gut Health of Growing Pigs. Animals.

[21] Zhang Y., Deng Z.X., He M.L., Pastor J.J., Tedo G., Liu J.X., Wang H.F. (2021). Olive oil cake extract stabilizes the physiological condition of lipopolysaccharide-challenged piglets by reducing oxidative stress and inflammatory responses and modulating the ileal microbiome. Food Funct..

[22] Zou C.Z., Zhao W.X., Yin S.G., Xiang X.Y., Tang J.Y., Jia G., Che L.Q., Liu G.M., Chen X.L., Tian G. (2024). Artificial parasin I protein (API) supplementation improves growth performance and intestinal health in weaned piglets challenged with enterotoxigenic Escherichia coli. Anim. Nutr..

[23] Meagher M.W., Johnson R.R., Young E.E., Vichaya E.G., Lunt S., Hardin E.A., Connor M.A., Welsh C.J.R. (2007). Interleukin-6 as a mechanism for the adverse effects of social stress on acute Theiler’s virus infection. Brain Behav. Immun..

[24] Stevenson L.S., McCullough K., Vincent I., Gilpin D.F., Summerfield A., Nielsen J., McNeilly F., Adair B.M., Allan G.M. (2006). Cytokine and C-reactive protein profiles induced by porcine circovirus type 2 experimental infection in 3-week-old piglets. Viral Immunol..

[25] Neurath M. (2017). Current and emerging therapeutic targets for IBD. Nat. Rev. Gastro Hepat..

[26] Jiang T.T., Gao X.J., Wu C., Tian F., Lei Q.C., Bi J.C., Xie B.X., Wang H.Y., Chen S., Wang X.Y. (2016). Apple-Derived Pectin Modulates Gut Microbiota, Improves Gut Barrier Function, and Attenuates Metabolic Endotoxemia in Rats with Diet-Induced Obesity. Nutrients.

[27] Xie L.M., Ge Y.Y., Huang X., Zhang Y.Q., Li J.X. (2015). Effects of fermentable dietary fiber supplementation on oxidative and inflammatory status in hemodialysis patients. Int. J. Clin. Exp. Med..

[28] Ding H.X., Zhang Q., Xu H.G., Yu X.N., Chen L.J., Wang Z.H., Feng J. (2021). Selection of copper and zinc dosages in pig diets based on the mutual benefit of animal growth and environmental protection. Ecotox. Environ. Safe.

[29] Huang J., Li S.K., Sung J.Y., Qiao S.Y., Zeng X.F., Zhou J.Y. (2025). Transfer of Antioxidant Capacity Through Placenta and Colostrum: β-Carotene and Superoxide Dismutase Collaboratively Enhance Integrated Breeding of Sows and Piglets. Antioxidants.

[30] Jin J.Q., Liu S.Y., Zhou Q., Fang Z.F., Lin Y., Xu S.Y., Feng B., Zhuo Y., Luo H.F., Liu X.M. (2025). Cinnamaldehyde supplementation in sows and their offspring: Effects on colostrum and milk composition, performance, redox status and intestinal health. J. Anim. Sci. Biotechno.

[31] Zeng X.L., Yang Y., Wang J.M., Wang Z.B., Li J., Yin Y.L., Yang H.S. (2022). Dietary butyrate, lauric acid and stearic acid improve gut morphology and epithelial cell turnover in weaned piglets. Anim. Nutr..

[32] Adesso S., Russo R., Quaroni A., Autore G., Marzocco S. (2018). Extract Attenuates Inflammation and Oxidative Stress in Intestinal Epithelial Cells via NF-κB Activation and Nrf2 Response. Int. J. Mol. Sci..

[33] Hermes R.G., Molist F., Ywazaki M., Nofrarías M., de Segura A.G., Gasa J., Pérez J.F. (2009). Effect of dietary level of protein and fiber on the productive performance and health status of piglets. J. Anim. Sci..

